# Elastic metasurfaces for Scholte–Stoneley wave control

**DOI:** 10.1098/rsta.2023.0365

**Published:** 2024-07-29

**Authors:** Farhad Zeighami, Said Quqa, Jacopo Maria De Ponti, Nadeen Ayyash, Alessandro Marzani, Antonio Palermo

**Affiliations:** ^1^ Department of Civil, Chemical, Environmental, and Materials Engineering—DICAM, University of Bologna, Viale del Risorgimento, 2, Bologna 40136, Italy; ^2^ Department of Civil and Environmental Engineering, Politecnico di Milano, Piazza Leonardo da Vinci, 32, Milano 20133, Italy

**Keywords:** Scholte–Stoneley waves, elastic metasurfaces, dispersion relation, graded metasurfaces

## Abstract

In this work, we investigate the dynamics of Scholte–Stoneley waves (SSWs) travelling along elastic metasurfaces, e.g. thin resonant structures embedding mechanical oscillators, placed at the interface between solid and fluid. To this purpose, an analytical dispersion law, valid in the long-wavelength regime, is derived and used to reveal the hybridization of SSWs with the collective resonance of the mechanical oscillators and the conversion of SSWs into leaky modes within the fluid. The analytical predictions are validated through numerical simulations that include both dispersive and harmonic analysis. Our findings disclose the capabilities of elastic metasurfaces in filtering, trapping and converting SSWs along fluid–solid interfaces, thus supporting the design of novel devices for solid–fluid interaction across various engineering applications, including microfluidics.

This article is part of the theme issue 'Current developments in elastic and acoustic metamaterials science (Part 1)'.

## 1. Introduction

Surface elastic waves are peculiar waves that propagate on the surface of elastic media and exponentially decay away from it. The most notable example is represented by Rayleigh waves [1], characterized by elliptical particle motions in the vertical plane, parallel to the direction of propagation.

The control of surface waves is of interest for several applications in both macro- and micro-scale domains. For this reason, elastic surfaces have been engineered through the addition of periodic and/or resonating structures to tailor the material dynamics. These structures, often referred to as elastic metasurfaces, allow the control of surface waves at a very small sub-wavelength scale, thanks to the hybridization of propagating waves with local resonance [2,3]. Notable applications include seismic isolation [3–7], sound absorbing [8,9], radio-frequency signal processing [10,11], imaging [12], non-destructive evaluation [13], sensing and energy harvesting [14]. More recently, elastic metasurfaces have been investigated considering spatial or temporal modulations. Arrays of resonators with spatially varying resonant frequency have been used to tailor surface and edge waves to achieve wave focusing [15,16], rainbow reflection, trapping, mode conversion [17,18] and topological states along plates, half-spaces [19] or lattices [20]. Control over the effective properties of elastic metasurfaces has also been obtained through quasiperiodic arrays in space [21] or time-modulated resonators [22–24].

In contrast to these studies, our focus lies in exploring elastic metasurfaces for the manipulation of a different type of surface waves, Scholte–Stoneley waves (SSWs), which propagate at the planar interface of a solid medium and a fluid [25,26].

An essential aspect regarding SSWs concerns the need for appropriate excitation methods. While SSWs can be reliably generated from the solid side through techniques such as laser-induced thermoelastic effects [27], laser-ultrasonic spectroscopy [28], impulsive stimulated thermal scattering [29] or by using piezoelectric materials, their excitation from plane acoustic waves in the fluid is not always feasible. Specifically, excitation may be prohibited if the dispersion relation of the surface wave falls below the sound cone, indicating that its phase velocity is lower than the velocity of waves in the fluid. This scenario typically occurs in SSWs propagating along a flat and smooth solid–fluid interface. Consequently, akin to the design principles governing spoof surface plasmon polaritons in photonics [30], the interface supporting SSWs is often modified with shallow corrugations [31] to match the differing crystal momentum of plane waves generated by acoustic transducers positioned in the surrounding fluid. SSWs can be generated through the diffraction of plane acoustic waves by a periodic grating, as periodicity can provide the additional reciprocal lattice wave vector necessary to satisfy the matching of wavevectors projected along the interface. However, this is not possible if diffraction results from the periodicity of the interface [32], as it was shown in deeply corrugated arrays of rectangular grooves in a silicon wafer [33] with propagating waves in water following the grating law.

Structured surfaces have also been patterned with phononic crystals at the solid–fluid interface [34]. For instance, directional gaps and band folding of SSWs have been achieved in phononic plates with in-plane periodic modulations of the elastic constants and smooth surfaces devoid of corrugations [32]. Periodic surface phononic crystals with corrugations [35] offer a range of interface waves, the characteristics of which are influenced by the depth of corrugation, enabling guidance or mode conversion. More recently, Umklapp diffraction has been proposed, in analogy with the purely elastic case [18], to focus underwater sound [36] through thin elastic plates submerged in water. Elastic metasurfaces consisting of graded arrays of resonators on fluid-loaded elastic plates [37] have also been used to mode-convert flexural waves into bulk acoustic waves and vice versa. Aside from structured interfaces, SSW also exists at the interface between ‘soft’ elastic plates in water. Notably, for soft materials, SSW presents a speed well below that of sound in water and is no longer mainly localized to the fluid [38].

Despite the abundance of available literature on SSWs, a complete analytical characterization of the interaction between SSWs and resonant arrays atop elastic half-spaces is still missing. While analytical formulations have been developed for fluid-loaded elastic plates [37] and fluid-filled slits with thermo-visco-elastic effects [39], fluid-loaded elastic half-spaces have been mainly analysed through the finite-element (FE) method, implementing variational formulations of the coupled elastic and acoustic wave propagation problem [33,35]. Hence, in this work, we derive analytical dispersion relations under long-wavelength approximation to describe the dynamic interaction between SSWs and elastic metasurfaces. Our analytical framework enables the characterization of hybridization of the fundamental mode around the collective resonant frequency and the exploration of phenomena such as surface wave leakage into the fluid domain, which depends on the thickness of the fluid layer.

The article is structured as follows: §2 presents the analytical derivation of the governing dispersion relation for SSWs propagating at the interface of a solid half-space and a fluid layer, decorated with elastic metasurfaces. In §3, the analytical dispersion law is discussed and validated through FE numerical simulations for two distinct scenarios: a fluid layer and a fluid half-space, overlaying a solid half-space equipped with vertical oscillators. In §4 we discuss, through harmonic numerical simulations, the dynamics of metasurfaces with frequency-variable resonators and their capabilities in manipulating the propagation of SSWs. Finally, §5 provides concluding remarks and discusses potential applications.

## 2. Dispersion analysis

**Figure 1.**
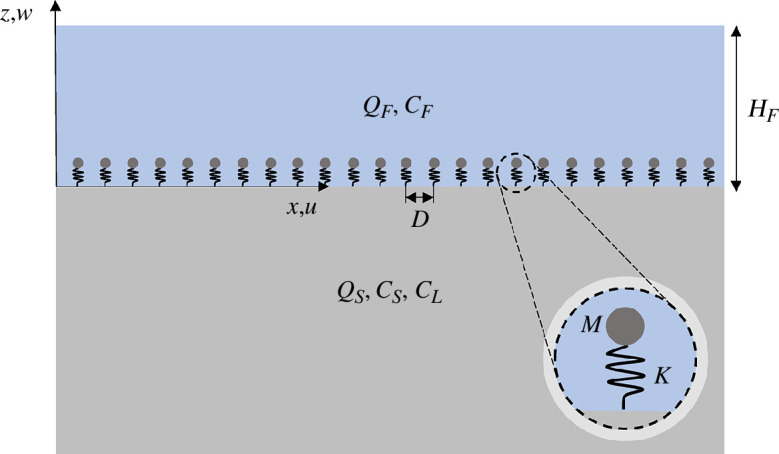
Schematic of a metasurface lying at the intersection between a solid half-space and a fluid layer.

In this section, we establish the dispersion equation for a metasurface interacting with SSWs. To achieve this, we focus our analysis on a plane-strain scenario, considering a system consisting of a solid half-space and a fluid layer. At the interface of these two mediums, we introduce an array of discrete mass-spring oscillators. The configuration of interest is shown in figure 1, where a periodic array of oscillators with a lattice distance D is considered [40–42].

### (a) Governing equation

#### (i) Solid half-space

The governing equation of motion in the elastic isotropic half-space can be written as


(2.1) $$(\lambda +\mu )\nabla (\nabla \cdot u)+\mu \nabla^{2}u=\rho_{S}\frac{\partial^{2}u}{\partial t^{2}},$$

with λ,μ being the Lamé constants, $u=[u_{x},u_{z}]$ the displacement vector, ∇=(∂/∂x,∂/∂z) the gradient operator, t represents time and $\rho_{S}$ the mass density. Following the Helmholtz decomposition u=∇Φ+∇×Ψ , the components of the displacement vector can be expressed as


(2.2) $$u_{x}=\frac{\partial \Phi }{\partial x}-\frac{\partial \Psi_{y}}{\partial z},\;\;\;\;u_{z}=\frac{\partial \Phi }{\partial z}+\frac{\partial \Psi_{y}}{\partial x},$$

in which Φ is a scalar potential, $\Psi_{y}$ is the y -component of the vector potential Ψ and x and z are the horizontal and vertical coordinates, respectively. Substituting equation (2.2) into equation (2.1) yields two uncoupled equations:


(2.3) $$\nabla^{2}\Phi =\frac{1}{c_{L}^{2}}\frac{\partial^{2}\Phi }{\partial t^{2}},\;\;\;\;\nabla^{2}\Psi_{y}=\frac{1}{c_{S}^{2}}\frac{\partial^{2}\Psi_{y}}{\partial t^{2}},$$

in which $c_{L}$ and $c_{S}$ are the velocities of pressure and shear waves in the solid, respectively, namely


(2.4) $$c_{L}=\sqrt{\frac{\lambda +2\mu }{\rho_{S}}},\;\;\;\;c_{S}=\sqrt{\frac{\mu }{\rho_{S}}}.$$

When harmonic surface waves propagating along the x -direction are sought, the potential functions can be chosen as


(2.5a) $$\Phi =A\;e^{\alpha z}\;e^{i(\omega t-kx)},$$

(2.5b) $$\Psi_{y}=B\;e^{\beta z}\;e^{i(\omega t-kx)},$$

where ω is the angular frequency, k is the wave number, A and B the amplitudes of the potentials and


(2.6) $$\alpha =\sqrt{k^{2}-\frac{\omega^{2}}{c_{L}^{2}}},\;\;\;\;\beta =\sqrt{k^{2}-\frac{\omega^{2}}{c_{S}^{2}}}.$$

Substituting into equation (2.5) and equation (2.2) yields the displacement components:


(2.7a) $$u_{x}=\left[-ik\left(Ae^{\alpha z}\right)-\beta \left(Be^{\beta z}\right)\right]e^{i(\omega t-kx)},$$

(2.7b) $$u_{z}=\left[\alpha \left(Ae^{\alpha z}\right)-ik\left(Be^{\beta z}\right)\right]e^{i(\omega t-kx)}.$$

The normal ( $\sigma_{zz,S}$ ) and tangential ( $\sigma_{zx,S}$ ) stress components in the solid are found according to the linear elastic, isotropic, constitutive law:


(2.8a) $$\sigma_{zz,S}=\lambda \frac{\partial u_{x}}{\partial x}+\left(\lambda +2\mu \right)\frac{\partial u_{z}}{\partial z}=\mu \left[\left(k^{2}+\beta^{2}\right)Ae^{\alpha z}+2ik\beta \left(-Be^{\beta z}\right)\right]e^{i(\omega t-kx)},$$

(2.8b) $$\sigma_{zx,S}=\mu \left(\frac{\partial u_{x}}{\partial z}+\frac{\partial u_{z}}{\partial x}\right)=\mu \left[2ik\alpha \left(-Ae^{\alpha z}\right)-\left(k^{2}+\beta^{2}\right)\left(Be^{\beta z}\right)\right]e^{i(\omega t-kx)}.$$

#### (ii) Fluid layer

Let us now consider the wave motion in the fluid. The governing equation, under the assumption of acoustic (inviscid) fluid, is


(2.9) $$\lambda_{F}\nabla (\nabla \cdot w)=\rho_{F}\frac{\partial^{2}w}{\partial t^{2}},$$

where $\lambda_{F}=\rho_{F}c_{F}^{2}$ is the adiabatic bulk modulus of the inviscid fluid, $\rho_{F}$ the fluid density, $c_{F}$ the fluid pressure wave velocity and $w=[w_{x},w_{z}]$ the fluid displacement vector. By introducing the scalar potential $\Phi_{F}$ , with $w=\nabla \Phi_{F}$ , equation (2.9) can be rewritten as


(2.10) $$\nabla^{2}\Phi_{F}=\frac{1}{c_{F}^{2}}\frac{\partial^{2}\Phi_{F}}{\partial t^{2}}.$$

Under harmonic wave propagation, the potential in the fluid layer is assumed as


(2.11) $$\Phi_{F}=(C_{1}\;e^{\gamma z}+C_{2}\;e^{-\gamma z})\;e^{i(\omega t-kx)},$$

where $C_{1}$ and $C_{2}$ are the potential amplitudes, while


(2.12) $$\gamma =\sqrt{k^{2}-\frac{\omega^{2}}{c_{F}^{2}}}.$$

Substituting equation (2.11) into $w=\nabla \Phi_{F}$ yields the displacement components:


(2.13a) $$w_{x}=\left[-ik\left(C_{1}e^{\gamma z}+C_{2}e^{-\gamma z}\right)\right]e^{i(\omega t-kx)},$$

(2.13b) $$w_{z}=\left[\gamma \left(C_{1}e^{\gamma z}-C_{2}e^{-\gamma z}\right)\right]e^{i(\omega t-kx)}.$$

In view of the fluid–solid coupling, we derive the fluid normal stress, which will later be used to compute the fluid pressure at the surface z=0 . Such a normal stress component can be derived as follows:


(2.14) $$\sigma_{zz,F}=\lambda_{F}\left(\frac{\partial w_{x}}{\partial x}+\frac{\partial w_{z}}{\partial z}\right)=\rho_{F}c_{F}^{2}(\gamma^{2}-k^{2})\left[C_{1}\;e^{\gamma z}+C_{2}\;e^{-\gamma z}\right]e^{i(\omega t-kx)}.$$

#### (iii) Mass-spring oscillator

To complete the formulation of the problem, let us now consider the dynamic equilibrium of a mass-spring resonator lying on the solid half-space surface:


(2.15) $$M\frac{d^{2}Z}{dt^{2}}+K\left(Z-u_{z,0}\right)=0,$$

where M is the resonator mass, K the spring stiffness, Z the vertical displacement of the resonator and $u_{z,0}$ the solid vertical displacement at the resonator base, namely, equation (2.7) at z=0 . Following the harmonic motion assumption, we express the resonator displacement Z as


(2.16) $$Z=\left(\frac{\omega_{r}^{2}}{\omega^{2}-\omega_{r}^{2}}\right)u_{z,0},$$

where $\omega_{r}=\sqrt{K/M}$ is the angular resonant frequency of the mass-spring system. By restricting our interest to the long-wavelength regime, namely, to wavelengths considerably larger than the array spacing D , we approximate the normal stress $\sigma_{zz,r}$ exerted by the resonator atop the solid surface ( z=0 ) as the resonator elastic force over its reference unit area $A_{r}$ :


(2.17) $$\sigma_{zz,r}(z=0)={\bar{\sigma }}_{zz,r}=\frac{M\omega_{r}^{2}}{A_{r}}\left(Z-u_{z,0}\right),$$

where $A_{r}=D^{2}$ for a square lattice and ${\bar{\sigma }}_{zz,r}$ is the averaged normal stress.

### (b) Dispersion relation

#### (i) Solid half-space with a fluid layer

Given the analytical descriptions of the wave motion in the solid half-space, fluid layer and resonators array, the SSW dispersion relation along the metasurface can be obtained by enforcing the following set of boundary conditions:


(2.18a) $$\begin{array}{c} \sigma_{zz,F}=0,\;\;\;\;\text{for}\;z=H_{F}, \end{array}$$

(2.18b) $$\begin{array}{c} \sigma_{zz,F}=\sigma_{zz,S}+\sigma_{zz,r},\;\;\;\text{for}\;z=0, \end{array}$$

(2.18c) $$\begin{array}{c} \sigma_{zx,S}=0,\;\;\;\;\;\text{for}\;z=0, \end{array}$$

(2.18d) $$\begin{array}{c} w_{z}=u_{z},\;\;\;\;\text{for}\;z=0. \end{array}$$

The inviscid fluid does not support shear stress and thus satisfies only the boundary condition in equation (2.18a) at its free surface $z=H_{F}$ . At the solid–fluid interface z=0 continuity of normal stress and vertical displacement is imposed (equations 18b and 18d). Notably, the normal stress in the solid accounts for the presence of the resonator. The set of boundary conditions is completed with zero shear stress imposed at the free surface of the solid (equation 18c). We remark that the above formulation neglects any direct kinematic coupling between the resonator and fluid. This simplification is justifiable when the dimensions of the resonator are significantly smaller than the wavelength of interest.

At this stage, by substituting equations (2. 7), (2.8), (2. 13), (2.14), (2.16) and (2.17) into the four boundary conditions equations (2.18a)–(2.18d), we obtain a system of four equations written in matrix form as


(2.19) $$\left[\begin{array}{cccc} 0 & 0 & e^{\gamma H_{F}} & e^{-\gamma H_{F}}\\ \mu (k^{2}+\beta^{2})+\alpha \Omega & -2\mu ik\beta -ik\Omega & -\rho_{F}c_{F}^{2}\left(\gamma^{2}-k^{2}\right) & -\rho_{F}c_{F}^{2}\left(\gamma^{2}-k^{2}\right)\\ -2ik\alpha & -\left(k^{2}+\beta^{2}\right) & 0 & 0\\ -\alpha & ik & \gamma & -\gamma \end{array}\right]\left[\begin{array}{c} A\\ B\\ C_{1}\\ C_{2} \end{array}\right]=0,$$

where


(2.20) $$\Omega \left(\omega \right)=\frac{M\omega_{r}^{2}\omega^{2}}{A_{r}\left(\omega^{2}-\omega_{r}^{2}\right)}$$

is the coupling factor between the solid half-space and the resonators.

Non-trivial solutions ( k,ω ) of equation (2.19) are found by imposing the determinant of the 4×4 matrix equal to zero, leading to the dispersion equation of SSW interacting with a metasurface placed at the interface of the fluid layer and solid half-space. As a closed-form solution of the system is not easily accessible, numerical schemes are adopted to compute the roots.

#### (ii) Solid and fluid half-space

We extend our analytical investigations to a configuration where the height of the fluid layer is significantly larger than the wavelength of interest, i.e. $kH_{F}\to \infty$ . In this scenario, the fluid can be modelled as a half-space and the related potential can be rewritten by assuming $C_{1}=0$ , thus avoiding an unbounded solution for z→∞ :


(2.21) $$\Phi_{F}=C_{2}\;e^{-\gamma z}\;e^{i\left(\omega t-kx\right)}.$$

Therefore, the related dispersion relation can be obtained by setting the reduced system of equations (2.18b)–(2.18d), whose matrix form reads


(2.22) $$\left[\begin{array}{ccc} \mu (k^{2}+\beta^{2})+\alpha \Omega & -2\mu ik\beta -ik\Omega & -\rho_{F}c_{F}^{2}\left(\gamma^{2}-k^{2}\right)\\ -2ik\alpha & -\left(k^{2}+\beta^{2}\right) & 0\\ -\alpha & ik & -\gamma \end{array}\right]\left[\begin{array}{c} A\\ B\\ C_{2} \end{array}\right]=0.$$

Non-trivial solutions ( k,ω ) of equation (2.22) are found by imposing the determinant of the 3×3 matrix equal to zero. With some algebra, a closed-form dispersion equation is obtained as


(2.23) $$\frac{\rho_{F}}{\rho_{S}}{\left(\frac{c}{c_{S}}\right)}^{4}\frac{\sqrt{1-\frac{c^{2}}{c_{L}^{2}}}}{\sqrt{1-\frac{c^{2}}{c_{F}^{2}}}}+{\left(2-\frac{c^{2}}{c_{S}^{2}}\right)}^{2}-4\sqrt{1-\frac{c^{2}}{c_{L}^{2}}}\sqrt{1-\frac{c^{2}}{c_{S}^{2}}}=\frac{\Omega c^{2}}{k\rho_{S}c_{S}^{4}}\sqrt{1-\frac{c^{2}}{c_{L}^{2}}},$$

where c=ω/k is the phase velocity of the SSW, which exhibits a dispersive behaviour ( c=c(ω) ).

As expected, when the presence of the fluid is neglected, i.e. $\rho_{F}=0$ , equation (2.23) recovers the dispersion of a simple solid half-space decorated with resonators [41]. The same equation provides the dispersion of the SSW at the interface between a solid and fluid half-space when M=0 (and thus, Ω=0 ), usually referred to in the literature as Brekhovskih’s formula [43]. For such a scenario, a single non-dispersive ( $c\left(\omega \right)=c_{SSW}$ ) wave mode is found with $c_{SSW}<c_{F}$ . We remark that a further highly decaying solution, the so-called leaky Rayleigh mode, exists in this configuration and radiates energy in the fluid owing to the condition $c_{LR}>c_{F}$ . Although an interaction between the leaky Rayleigh mode and the metasurfaces is expected, its investigation falls beyond the scope of this work.

## 3. Case studies

In this section, we numerically investigate the dispersion laws of SSWs travelling along a solid–fluid interface decorated with a metasurface of periodically arranged vertical oscillators, as obtained in the previous section. To generalize our analysis, we introduce the following normalized quantities:

— velocities: $c_{F}^{\prime }=\frac{c_{F}}{c_{F}}$ , $c_{S}^{\prime }=\frac{c_{S}}{c_{F}}$ , $c_{L}^{\prime }=\frac{c_{L}}{c_{F}}$ ;
— frequency and wavenumber: $\omega^{\prime }=\frac{\omega }{\omega_{r}}$ , $k^{\prime }=\frac{k}{k_{r}}$ , with $k_{r}=\frac{\omega_{r}}{c_{F}}$ ;
— normalized mass and density: $\rho_{F}^{\prime }=\frac{\rho_{F}}{\rho_{F}}$ , $\rho_{S}^{\prime }=\frac{\rho_{S}}{\rho_{F}}$ , $M^{\prime }=\frac{Mk_{r}}{A_{r}\rho_{F}}$ ;
— normalized coordinates and length: $x^{\prime }=xk_{r}$ , $z^{\prime }=zk_{r}$ , $H_{F}^{\prime }=H_{F}k_{r}$ , $D^{\prime }=Dk_{r}$ .

The normalized mechanical and geometrical parameters used in the case studies are listed in table 1. The solid and fluid material values are chosen to fulfill the condition $c_{S}>c_{F}$ , resulting in a standard Scholte wave speed close to the fluid velocity. Two distinct layer heights are considered, namely, $H_{F}^{\prime }$ = [ π , ∞ ], which represent a fluid layer and half-space scenarios, with their dispersion relations provided in equations (2.19) and (2.23), respectively. The analysis is conducted by computing the dispersion curves according to the developed formulation, leveraging the Newton–Raphson method. The numerical roots of the dispersion relations are obtained by starting with values of $\omega^{\prime }$ and calculating the real part of the complex dimensionless wavenumber $k^{\prime }$ [44]. The validity of both the dispersion relations and the adopted root-finding scheme is confirmed through numerical FE simulations.

**Table 1.** Mechanical and geometrical parameters of the metasurface, solid half-space and fluid layer.

normalized parameter	symbol	value
longitudinal wave velocity solid	$c_{L}^{\prime }$	3.5
shear wave velocity solid	$c_{S}^{\prime }$	1.5
mass density of solid	$\rho_{S}^{\prime }$	1.5
resonator mass	$M^{\prime }$	0.5
fluid layer thickness	$H_{F}^{\prime }$	π , ∞
lattice spacing	$D^{\prime }$	0.5

### (a) Solid half-space and fluid layer

**Figure 2.**
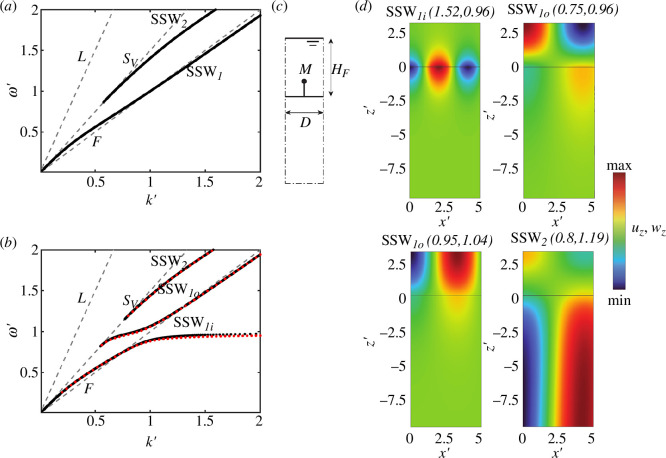
Dispersion relation of SSW propagating at the interface of a fluid layer attached to a solid half-space (*a*) without and (*b*) with metasurfaces following table 1. The red dots superimposed on the figure represent the numerical dispersion curves. (*c*) Numerical model of the unit cell to verify the analytical dispersion law. (*d*) Vertical displacement wavefields of selected mode shapes.

On the one hand, figure 2*a* reports the dispersion curve of SSWs when no resonators are placed over the solid surface, as obtained from equation (2.22) with $M^{\prime }=0$ and $H_{F}^{\prime }=\pi$ . On the other hand, figure 2*b* shows the dispersion curve ( $\omega^{\prime }$ versus $k^{\prime }$ ), as obtained from the solution of equation (2.22) with resonators following table 1. In both figures, black dots are used to mark the SSW modes while grey dashed lines are used to label bulk modes in the solid (longitudinal (L) and shear (S)) and in the fluid (F).

Before delving into the dynamics of the metasurface, it is worth recalling the fundamental dispersive feature of Scholte–Stoneley modes supported by a finite thick fluid layer. As evident from figure 2*a*, SSWs are dispersive and multimodal. In the low-frequency regime, the fundamental SSW_1_ mode propagates with a phase velocity $c\approx c_{S}$ . The velocity decreases at higher frequencies approaching the velocity $c_{SSW}$ obtained as a solution of equation (2.23). The next $n^{\text{th}}$ mode appears at the cut-on frequency:


(3.1) $$\omega_{c,n}=\frac{tan^{-1}\left(\frac{-\rho_{S}\sqrt{\frac{c_{S}^{2}}{c_{L}^{2}}-1}}{\rho_{F}\sqrt{1-\frac{c^{2}}{c_{F}^{2}}}}\right)+n\pi }{H_{F}\sqrt{\frac{1}{c_{F}^{2}}-\frac{1}{c_{S}^{2}}}}.$$

Let us now shift our interest to the metasurface dispersion curve (figure 2*b*); red dots are used to label the numerical solutions obtained through FE models. We develop a two-dimensional (2D) FE model of the unit cell using Comsol Multiphysics® [45], exploiting the wave FE approach [46], as shown in figure 2*c*. The unit cell has a normalized width of $D^{\prime }$ and a height of $10\;H_{F}^{\prime }$ for the solid domain. The normalized height of the fluid domain is $H_{F}^{\prime }=\pi$ . The fluid is modelled as a compressible lossless (no thermal conduction and no viscosity) medium satisfying the Helmholtz equation, while the solid is a linear elastic material. The mechanical properties are those listed in table 1. The resonators are modelled as a discrete mass-spring system, with trusses and point masses at the tips of the truss elements. The acoustic-structure boundary coupling is used at the interface of the solid half-space and fluid layer. The coupling includes the fluid load on the solid domain and allows the transfer of stresses between the two domains (more details are provided in COMSOL [47]).

Quadrilateral elements with a maximum mesh size of $D^{\prime }/2$ are used to discretize the solid and fluid domains, while single truss elements are used to discretize the oscillators. To avoid any undesirable rigid-body motion of the unit cells, the base displacements of the cell are fixed. In the unit cell model, SSW propagates in the $x^{\prime }$ -direction, therefore, periodic (Bloch) boundary conditions are imposed parallel to the wave propagation direction to its lateral edges. The eigenfrequency analysis is performed by sweeping the wavenumber $k^{\prime }$ within the range $k^{\prime }=[0.2,2]$ . The accuracy of this approach has been verified in previous works [5,48]. The reader can appreciate the excellent agreement between the analytic (black dots) and FE numerical predictions (red dots) shown in figure 2*b*. The numerical SSW modes are identified through numerical simulations by considering only the modes that satisfy $c<c_{S}$ . We remark that solutions in the low-frequency range ( $\omega^{\prime }<0.2$ ) are not sought, as their long wavelengths would necessitate a numerical model thickness sufficiently large to accurately capture the fundamental SSW mode.

Moving to the investigation of the metasurface dispersion, we observe how the resonance of the vertical oscillators hybridizes with the fundamental SSW_1_ mode giving rise to two distinct and repelling branches: a slow mode SSW_1*i*_ which asymptotically approaches the oscillator resonance and a second branch, SSW_1_ *_o_* with a cut-on frequency below the oscillator resonance, approaching the standard SSW_1_ mode for $\omega^{\prime }>1$ . As for the other modes, the cut-on frequency is found at the crossing point with the shear vertical ( $S_{V}$ ) mode of the solid. Notably, no frequency band gap arises owing to the presence of the surface oscillators, in contrast with what is typically observed for surface waves propagating along a metasurface of vertical oscillators [41]. Regarding the SSW_2_ mode, besides a slight shift of the mode cut-on frequency, no significant influence owing to the presence of the oscillator is found.

Further details on the metasurface dynamics are found by inspecting the SSW eigensolutions. To this purpose, the vertical displacement profile within solid $\left(u_{z}\right)$ and fluid ( $w_{z}$ ) layers, for selected mode shapes in the frequency range close to the oscillator resonance, are shown in figure 2*d*.

For the SSW_1*i*_ at [ $k^{\prime }=1.52$ , $\omega^{\prime }=0.96$ ], the wave motion in both the fluid and the solid layer is confined along the metasurface owing to the hybridization with the localized oscillator resonance. Notably, the displacement of the solid free surface is in phase with the vertical motion of the resonator. Conversely, along the upper branch SSW_1_ *_o_*, see ([ $k^{\prime }$ , $\omega^{\prime }=0.96$ ] and [ $k^{\prime }=0.95$ , $\omega^{\prime }=1.04$ ]), the motion remains mostly confined within the fluid layer. This could be ascribed to the effect of the metasurface on the solid–fluid interface which acts as rigid impedance for the fluid. For completeness, we also report the wave motion associated with the SSW_2_ ([ $k^{\prime }=0.8$ , $\omega^{\prime }=1.19$ ]), which extends all over the mapped domain. The reader may verify that this wave motion corresponds to the one of a standard SSW_2_ mode.

### (b) Solid and fluid half-spaces

**Figure 3.**
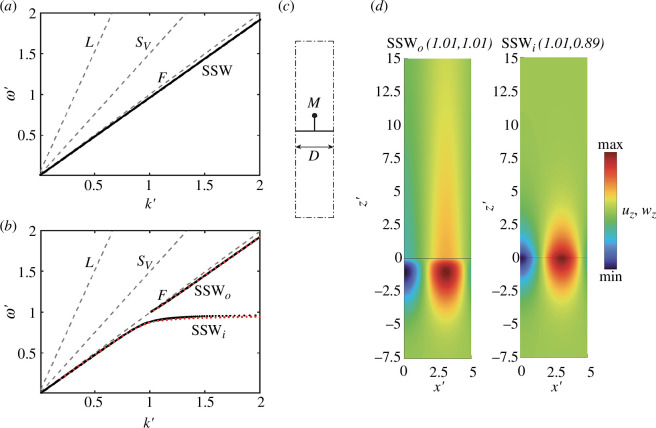
Dispersion relation of SSWs propagating at the interface of a fluid half-space attached to a solid half-space (*a*) without and (*b*) with metasurfaces following table 1. (*c*) Numerical model of the unit cell to verify the analytical dispersion law. The red dots superimposed on the figure represent the numerical dispersion curves. (*d*) Vertical displacement wavefields of selected mode shapes.

Let us now focus our attention on the scenario where the metasurface is placed at the intersection of two half-spaces, solid and fluid or simply when the wavelength of interest is much smaller than the thickness of the fluid layer ( $H_{F}k\to \infty$ ). The dispersion curve ( $\omega^{\prime }$ versus $k^{\prime }$ ) obtained from the solution of equation (2.23) in the cases M=0 and M≠0 is shown in figure 3. Again, black dots are used to mark the SSW modes, grey dashed lines to label bulk modes in the solid (longitudinal (L) and shear (S)) and in the fluid (F), and red dots for the numerical solutions. The latter are obtained using a unit cell model with a fluid layer thickness $H_{F}^{\prime }=10\pi$ , schematically shown in figure 3*c*, and considering specifically SSW modes where the phase velocity is less than the fluid phase velocity $c<c_{F}$ . The reader may again appreciate the excellent agreement between numerical and analytical solutions.

As observed for the finite-thickness fluid layer, the hybridization phenomenon between the fundamental SSW and the resonance yields two repelling branches. In more detail, the higher branch SSW*_0_* sets out from a cut-on frequency which lies exactly at the oscillator resonance. The crossing point marks the transition between a confined mode travelling along the half-space intersection and a mode leaking its energy into the fluid bulk. The wave motion in this transition point is mapped in figure 3*d* where the extended wavefield in the fluid at [ $k^{\prime }=1.01$ , $\omega^{\prime }=1.01$ ] is displayed. The reader can compare it with the confined mode SSW_*i*_ at [ $k^{\prime }=1.01$ , $\omega^{\prime }=0.89$ ].

## 4. Application: SSW trapping and conversion through graded metasurface

**Figure 4.**
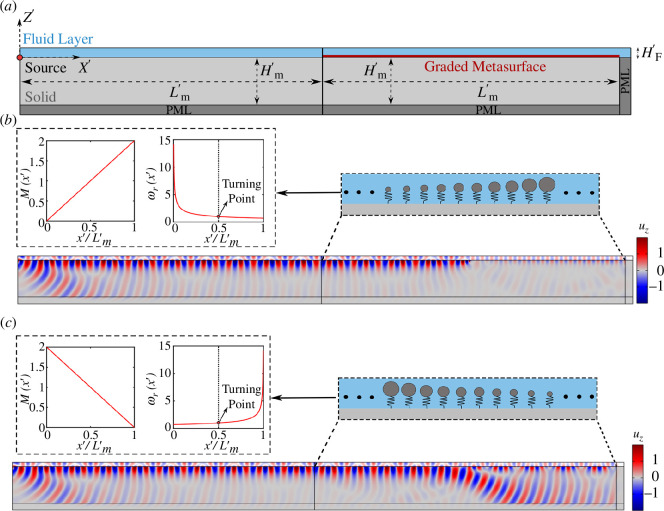
(*a*) Schematic of the 2D FE model of graded metasurfaces used in the harmonic analysis. Graded resonators with linearly (*b*) increasing and (*c*) decreasing mass over the metasurface array form the graded metasurface. The insets of (*b*) and (*c*) show the variation of mass and resonant frequency of the graded resonators. The normalized pressure field of the fluid layer along with the vertical displacement of solid half-space for a harmonic excitation at $\omega^{\prime }=1$ are shown for both cases.

**Figure 5.**
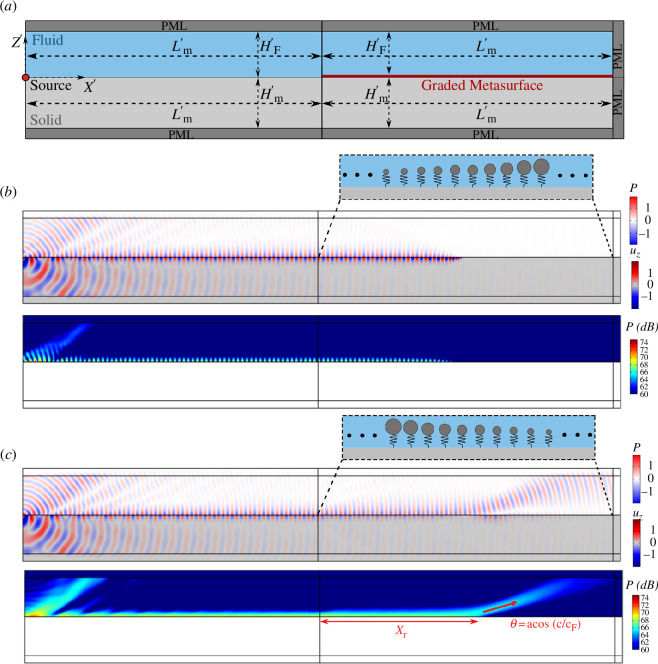
(*a*) Schematic of the 2D FE model of graded metasurfaces accommodated at the interface of a solid–fluid half-spaces. (*b*) The normalized pressure field of the fluid half-space and the vertical displacement of the solid half-space for a harmonic excitation at $\omega^{\prime }=1$ of graded metasurface with increasing mass (top panel). The acoustic pressure level (dB) is shown at resonance (bottom panel). (*c*) The same quantities are shown for the graded metasurface with decreasing mass.

In this section, we use the discussed dispersion laws for the design of a graded metasurface capable of controlling the propagation of SSWs. Specifically, herein, we adopt a 2D FE model, comprising a solid half-space attached to a fluid layer, as depicted in figure 4*a*. Owing to symmetry, only half of the model is shown. We consider two distinct sections: (i) a buffer zone where a point source generates SSWs along a pristine solid–fluid interface; and (ii) a metasurface zone where the graded resonators are allocated to manipulate the propagation of SSWs along the $x^{\prime }$ -direction. Each solid domain has normalized dimensions of $L_{m}^{\prime }\times H_{m}^{\prime }$ , where $H_{m}^{\prime }=10\pi$ and $L_{m}^{\prime }=nD^{\prime }$ , in which n=400 is the number of resonators. For the fluid layer, two distinct heights are considered, namely, $H_{F}^{\prime }=[\pi ,10\pi ]$ , the latter being representative of a half-space scenario, as shown schematically in figure 5*a*. In both scenarios, the harmonic source is positioned at a distance of $L_{m}^{\prime }$ from the edge of the metasurface zone to reduce near-source effects, and the perfectly matched layers (PMLs) are allocated at the boundaries of the solid and fluid domains to minimize back-reflections. The bottom edge of the model is restrained to avoid any unwanted rigid motion during the simulation. The metasurface is formed by replicating the FE model of the unit cell, discussed in the previous section, in the $x^{\prime }$ -direction. The same mesh type and size of the unit cell are used to discretize the full model.

As the first case study, we examine a fluid layer with a height of $H_{F}^{\prime }=\pi$ . Following the design methodology adopted in Colombi *et al.* [17], we consider graded metasurfaces with either (i) decreasing or (ii) increasing resonant frequency along the array. For the former, we consider a linearly increasing resonator mass along the metasurface while maintaining the stiffness of the springs constant. This results in a resonant frequency versus position law $\omega_{r}\left(x^{\prime }\right)\propto x^{\prime -\frac{1}{2}}$ , as depicted in the inset of figure 4*b*. Notably, we set $\omega_{r}(L_{m}^{\prime }/2)$ as the reference resonance value for computing all the normalized quantities.

The propagation of SSW along a metasurface with decreasing resonant frequency is analysed for a harmonic excitation at $\omega^{\prime }=1$ , namely $\omega =\omega_{r}(L_{m}^{\prime }/2)$ . Figure 4*b* shows the related normalized vertical displacement field within the solid medium and the normalized acoustic pressure field in the fluid layer. The SSWs propagate undisturbed until they reach the metasurface region, where they experience a progressive localization owing to the interaction with the graded configuration. Along the array, the wave propagates as a hybridized surface mode of the type SSW_1*i*_. As the mass of the resonators increases, the group velocity of the wave gradually decreases until it reaches the location $x^{\prime }=L_{m}^{\prime }/2$ , where the wave frequency matches the resonator frequency. At this location, commonly labelled as the turning point, the wave energy in the solid strongly localizes, following the rainbow-effect phenomenon also observed for Rayleigh waves [17]. Notably, a small portion of the wave energy continues to propagate along the array, mostly confined within the fluid layer. This can be possibly ascribed to the non-adiabatic variation of frequencies along the metasurface array which leads to a small energy exchange between the SSW_1*i*_ and the SSW_1_ *_o_*. Indeed, these two modes are not separated by a frequency gap (see figure 2*b*).

For the second configuration, we instead consider an array with linear-decreasing resonator mass and constant stiffness. This results in the resonant frequency versus position law $\omega_{r}(x^{\prime })\propto {\left(1-x^{\prime }\right)}^{-\frac{1}{2}}$ , as shown in the inset of figure 4*c*. Differently from the previous scenario, here the SSW propagation along the metasurface is driven by the upper hybridized branch of the dispersion curve (SSW_1_ *_o_*). Owing to the variation of resonant frequency along the array, the mode experiences a gradual delocalization from the surface up to the turning point position $x^{\prime }=0.42\;L_{m}^{\prime }$ , where the wave frequency matches the upper branch cut-on frequency, namely the wave phase velocity $c_{SSW_{1o}}=c_{S_{V}}$ . Note that this frequency is slightly lower than the resonance (refer to figure 2*b*); as such, the turning point is slightly shifted from the metasurface midpoint. Since $c_{SSW_{1o}}=c_{S_{V}}$ , the SSW mode is diverted into a bulk shear mode which leaks its energy into the medium. Again, beyond the turning point, part of the wavefield continues to propagate confined along the surface, possibly owing to the coupling between the SSW_1_ *_o_* and the SSW_1*i*_ modes.

We replicate our analysis considering a fluid half-space or equivalently a fluid domain with sufficiently large height resembling a semi-infinite scenario ( $H_{F}^{\prime }=10\pi$ ). A schematic of the numerical model is shown in figure 5*a*. In contrast with the previous model, PMLs are here adopted along the external boundaries of the fluid domain to mimic an unbounded medium. Again, two metasurface configurations with linearly increasing and decreasing mass over the array are considered; refer to figure 5*b,c*, respectively. In addition to the vertical displacement and the acoustic pressure field, the sound pressure level (dB) in the fluid is also computed for both scenarios.

For a frequency-decreasing metasurface (figure 5*b*), the wave propagation phenomenon resembles what was previously observed in the fluid-layer configuration: the hybridized SSW_*i*_ is smoothly localized by the graded metasurface. The wave terminates at the turning point $x^{\prime }=L_{m}/2^{\prime }$ where the wave frequency matches the resonator frequency. Differently from the fluid-layer scenario, no evidence of coupling with higher-order modes is found beyond the turning point, where the wavefield becomes almost negligible.

Conversely, for frequency-increasing metasurface (figure 5*c*), the interface wave propagates along the array as an SSW_*0*_ mode which is smoothly converted into a bulk mode travelling in the interior of the fluid domain. The mode conversion occurs again at the turning point $x^{\prime }=L_{m}^{\prime }/2$ , namely, where the frequency of the wave matches the cut-on frequency of the mode. As anticipated in §3, the cut-on frequency of the SSW_*0*_ mode marks the transition between a confined mode travelling along the half-space intersection and a mode leaking its energy into the fluid bulk. Notably, the inclination angle of the mode generated within the fluid can be estimated using Snell’s law as $\theta =acos\left(c/c_{F}\right)$ .

## 5. Conclusions

We investigated the dynamics of SSWs propagating along the interface of a fluid layer attached to a solid half-space equipped with an elastic metasurface. The metasurface comprises an array of periodically distributed discrete mass-spring resonators with sub-wavelength dimensions and spacing. We derived an analytical dispersion law, valid in the long-wavelength regime, that predicts the interaction between the vertical resonators and the SSW modes. The dispersion law evidences the hybridization phenomenon between the fundamental SSW mode and the resonance of the oscillators. Notably, the hybridization does not generate a frequency bandgap, as typically observed for surface Rayleigh waves interacting with vertical oscillators. Next, we extended our analysis to an unbounded fluid domain. In this case, the dynamic interaction of elastic metasurfaces and SSW resulted in the splitting of the fundamental SSW mode into two repelling branches, exhibiting a classical avoided-crossing behaviour. The cut-on frequency of the upper branch marks the transition point between surface-confined waves and waves leaking into the fluid.

The analytical findings were validated through numerical FE simulations, confirming the accuracy of the derived dispersion laws. A thorough harmonic analysis further highlighted the underlying physics of the problem. Specifically, by leveraging the dispersion, we designed graded metasurfaces with both increasing and decreasing resonant frequencies. For the case of graded metasurface with decreasing resonant frequencies, the localization of SSWs at the fluid–solid interface was observed. Conversely, in the case of graded metasurface with increasing resonant frequencies, the SSW-to-shear conversion and the SSW leakage as a bulk wave in the fluid were observed.

In summary, the proposed model is a powerful tool for designing metasurfaces to control SSWs, paving the way towards innovative applications in surface acoustic wave microfluidics. These applications include fluid mixing, translation, particle jetting and sorting. To enhance the presented model, future work should extend the analytical formulation by incorporating more advanced features, such as realistic rheological fluid laws and anisotropic solid materials.

## Data Availability

The authors confirm that the data supporting the findings of this study are available within the article.
